# A CRISPR-Cas9 Gene Drive System Targeting Female Reproduction in the Malaria Mosquito vector *Anopheles gambiae*

**DOI:** 10.1038/nbt.3439

**Published:** 2015-12-07

**Authors:** Andrew Hammond, Roberto Galizi, Kyros Kyrou, Alekos Simoni, Carla Siniscalchi, Dimitris Katsanos, Matthew Gribble, Dean Baker, Eric Marois, Steven Russell, Austin Burt, Nikolai Windbichler, Andrea Crisanti, Tony Nolan

**Affiliations:** 1 Department of Life Sciences, Imperial College London, SW7 2AZ, UK; 2 Dipartimento di Medicina Sperimentale Via Gambuli, Centro di Genomica Funzionale, University of Perugia, 06132 Perugia, Italy; 3 Department of Genetics, University of Cambridge, CB2 3EH, UK; 4 INSERM U963, CNRS UPR9022, Université de Strasbourg, Institut de Biologie Moléculaire et Cellulaire, 67084 Strasbourg, France

## Abstract

Gene-drive systems that enable super-Mendelian inheritance of a transgene have the potential to modify insect populations over a timeframe of a few years [AU please provide a real estimate, this seems vague]. We describe CRISPR-Cas9 endonuclease constructs that function as gene-drive systems in *Anopheles gambiae*, the main vector for malaria [AU:OK?]. We identified three genes (*AGAP005958*, *AGAP011377* and *AGAP007280*) that confer a recessive female sterility phenotype upon disruption, and inserted into each locus CRISPR-Cas9 gene-drive constructs designed to target and edit each gene [AU:OK?]. For each locus targeted we observed strong gene drive at the molecular level, with transmission rates to progeny of 91 to 99.6%. Population modelling and cage experiments indicate that a CRISPR-Cas9 construct targeting one of these loci, *AGAP007280,* meets the minimum requirement for a gene drive targeting female reproduction in an insect population. These findings could expedite the development of gene drives to control suppress mosquito populations to levels that do not support malaria transmission.

Synthetic gene-drive systems using site-specific endonucleases to spread traits into a population were first proposed more than a decade ago 1. This proposal was initially inspired by the action of a class of natural selfish genetic element, found in many single cell organisms, named Homing Endonuclease Genes (HEGs). HEG-encoded proteins can recognise and cleave a 15-30bp DNA sequence. HEGs are located within the DNA recognition sequence, rendering it resistant to further cleavage. However when the HEG comes into contact with a chromosome containing the uninterrupted recognition sequence the double stranded break induced by the cleavage is often repaired using the homologous chromosome as a template, effectively converting a heterozygote into a homozygote in a process known as ‘homing’. Through this mechanism, the frequency of an HEG can rapidly increase in a population. Naturally occurring HEGs can in principle be adapted to function as a gene drive system in mosquitoes because they can be re-engineered to recognise mosquito genes^2^. An HEG expressed in the male mosquito germline that recognises an artificially introduced recognition site shows high rates of super-Mendelian inheritance and rapidly invades a caged population 2. The increased transmission rate provided by endonuclease-based gene- drive systems could theoretically outweigh the fitness costs arising from the cleavage activity and disruption of the targeted sites. If this provise is met, a drive construct can spread through a population until it reaches an equilibrium frequency, with a reduced mean fitness for the population 1.

Any nuclease with a sufficiently long recognition sequence could hypothetically be re-designed to function as a gene drive system akin to an HEG, provided that it can be engineered to recognise and insert in a specific genomic locus. For example, we have previously shown that modular nucleases such as zinc finger nucleases or TALENs, for which the DNA-binding specificity of each module is well characterised, can be combined to function as a synthetic selfish element in *Drosophila*, albeit with low replication fidelity owing to their repetitive nature 3. More recently, the development of the CRISPR/Cas9 system 4–6 has radically simplified the process of engineering nucleases that can cleave specific genomic sequences. A guide RNA (gRNA) complementary to a DNA target site directs the activity of the Cas9 endonuclease to that sequence, providing a means to edit almost any chosen DNA sequence without the need to undertake complex protein engineering and selection procedures. In addition to applications in genome editing, the specificity and the flexibility of the CRISPR/Cas9 system offers unprecedented opportunities to expedite the development of gene-drive systems for the control of insect vectors of disease 7. In a proof of principle for such a use, a CRISPR based construct was used to demonstrate gene drive in a single generation at an eye colour locus in both *Drosophila* and yeast using a split drive system 8,9.

Translation of this technology for the control of the insect vector of human malaria requires development of an endonuclease-based gene drive system that interferes with the ability of *A. gambiae* mosquitoes to transmit the disease. This could be achieved either by blocking parasite development or by reducing the reproductive capability of the insect vector. Modelling of vector populations indicates that the latter might be achieved through the use of an endonuclease designed to ‘home’ and yield a recessive mutation in a gene that is essential for viability or female fertility, with the latter being more effective provided that homing is temporally and spatially confined to the germline during, or prior to, the process of gamete formation 1,10. This is essential to avoid somatic disruption of the wild type allele and allow the normal development of heterozygous mosquitoes, critical for transmitting the endonuclease to the subsequent generations. Along these lines we have developed a CRISPR-based gene drive system designed to perform homing in both sexes of the human malaria vector *A. gambiae*, targeting haplosufficient somatically expressed female fertility genes.

To identify putative female fertility genes in *A. gambiae* we used a combination of orthology and a sterility index based on a logistic regression model that correlated gene expression features with likelihood of female sterile alleles in the model dipteran *Drosophila melanogaster* 11,12. Three candidate genes with high ovary expression and tissue-specificity were chosen from this analysis: *AGAP005958* (ortholog of *Drosophila yellow-g*, a haplosufficient female fertility gene expressed in somatic follicle cells 13); *AGAP007280* (ortholog of *Drosophila nudel*, a haplosufficient female fertility gene expressed in somatic follicle cells involved in dorsoventral patterning of embryo 14); *AGAP011377* (no apparent *Drosophila* ortholog but contains a probable chitin binding domain).

We used either CRISPR or TALEN nucleases to selectively disrupt the coding sequence of these candidate genes and analysed reproductive phenotypes to validate the suitability of these genes as homing targets. The gene knockout strategy generated “docking lines” via homologous recombination inserting a GFP transcription unit flanked by two attP sites suitable for subsequent insertion of active drive constructs (Fig 1A). Though not strictly necessary for the purpose of inserting a gene drive element, the generation of the docking lines allows an unambiguous assessment of the phenotype caused by gene disruption, in the absence of ongoing Cas9 activity. In each case the insertion of the attP–GFP docking cassette was designed to produce a null phenotype. Both TALEN and Cas9 nucleases were effective in cutting the corresponding target sequences and in promoting the insertion of the docking construct at the cleavage site. At each of the three selected target loci transformed GFP+ individuals were recovered at a relatively high frequency with rates at least comparable to our experience of transposon-mediated germline transformation (Supp Table 1) and they were confirmed in PCR experiments to carry the desired homologous recombination events (See Supp. Fig 1). **[AU needs to be made clear whether your method REQUIRES a docking line to be constructed, in the text please]** . G_1_ individuals of the docking lines were fertile and were intercrossed to produce G_2_ progeny, expected to include individuals both heterozygous and homozygous for the insertion. Visual inspection of G_2_ progeny identified two classes of mosquitoes on the basis of GFP intensity, ‘intermediate’ and ‘strong’, which we attributed to the presence of one or two copies of the GFP gene in the heterozygous or homozygous state respectively, and later confirmed by molecular analysis (Fig 1B). Fertility assays (egg laying and hatching) performed on individual mosquitoes showed that all homozygous female mosquitoes were sterile whereas heterozygous females showed normal rates of egg laying and hatching (Fig 1C). On the basis of these results we concluded that the selected genes should be regarded as haplosufficient female sterility genes. Manifestation of the impaired fertility phenotype differed across the 3 genes targeted, consistent with their function at distinct stages of egg production and embryo development: homozygous females carrying two disrupted alleles of either *AGAP005958* or *AGAP011377* failed to lay eggs whereas homozygous mutant females at *AGAP007280* laid eggs that did not hatch (see Supp. Fig 2).

After validating the female fertility phenotype of the target genes we inserted a gene-drive element (*CRISPR^h^*) (Fig 1a) into the docking site by recombinase mediated cassette exchange (RMCE) 15. Each drive construct was designed to home in both sexes into the cognate wild type locus and contained the following elements: a) the Cas9 nuclease under the control of the *vasa2* promoter, shown in a previous report to be active in the germline of both sexes 16; b) a gRNA sequence designed to direct the cleavage activity of the nuclease to the same sequence targeted in the gene knockout experiments and under the promoter of the ubiquitously expressed, PolIII-transcribed *U6* gene 17; and c) a visual marker (3xP3::RFP). AttB sites flanking the *CRISPR^h^* construct were used to direct ФC31 integrase-mediated recombination at the docking site. Aware of the potential for these mosquitoes to show gene drive, we housed our mosquitoes in a containment facility consistent with recent recommendations for safeguards in such experiments 18. Successful cassette exchange events were visually identified among G_1_ progeny as GFP+ to RFP+ phenotype conversions, and confirmed using PCR (Supp. Fig 3). [AU all primer sequences must be included in your final paper]. At all three female fertility loci we recovered double crossover events that resulted in cassette exchange and insertion of the *CRISPR^h^* allele, at transformation frequencies ranging from 2 to 7% (Table 1). In the cassette exchange reaction we observed the insertion of both complete and incomplete *CRISPR^h^* alleles, the latter probably the result of intramolecular recombination between regions of homology between the guide RNA construct and its endogenous target at the insertion site.

Each complete integration event generated a *CRISPR^h^* modified allele encoding a Cas9-gRNA endonuclease designed to target the corresponding integration site on a wild type chromosome. Accordingly the *CRISPR^h^* allele was resistant to nuclease cleavage as its target sequence had been interrupted by the insertion of the CRISPR^h^ construct itself. In heterozygous mosquitoes the activation of the *vasa2* promoter during gamete formation should induce the synthesis of the Cas9 nuclease that in concert with the ubiquitously expressed gRNA should cleave the target sequence in the fertility genes, thereby initiating homologous recombination repair events that lead to the homing of the CRISPR^h^ construct into the wild type allele (Fig 2A). Visual screening was used to analyse the frequency of the RFP-linked *CRISPR^h^* allele in the progeny of heterozygous parents crossed to wild type mosquitoes to detect signs of non-Mendelian inheritance above the expected frequency of 50% that would reveal gene drive activity (Fig 2B).

In several of the *CRISPR^h^/+* G_1_ individuals that we recovered at each locus we noticed super-Mendelian inheritance of the RFP-marked *CRISPR^h^* allele [AU briefly say how this was obvious], with rates as high as 94.4 -100% (Table 1), among the progeny. To further investigate the activity of these *CRISPR^h^* alleles we looked at the homing ability and sterility in the G_2_ generation and beyond, scoring the progeny of large numbers of single crosses to wild type mosquitoes. Invariably we saw high rates of transmission in every fertile cross examined (Fig 2C), representing average homing rates (defined as the proportion of non- CRISPR alleles converted to *CRISPR^h^* in the gametes) ranging from 87.3% to 99.3% across the 3 target genes. Importantly, though we observed more variability (69-98%) across generations we observed no obvious decrease in homing performance over time (Table 2), suggesting that the majority of CRISPR homing events regenerate an intact allele. Furthermore the transmission rate of the *CRISPR^h^* allele at AGAP007280 and AGAP011377 was high in both male and female *CRISPR^h^/+* individuals, in agreement with the predicted activity of the *vasa2* promoter in both sexes during early gametogenesis (Papathanos et al. 2008). In those rare progeny that did not contain a CRISPR homing allele, we looked for evidence of repair by non-homologous end joining (NHEJ), microhomology-mediated end joining (MMEJ)^19^ or other non-canonical homing events at the three target loci. From a total of 32 offspring derived from a minimum of 7 individuals we found a total of 12 indel mutations (6 unique, including two examples of a 6bp deletion that preserves reading frame and could represent a resistant allele), presumably arising from NHEJ or MMEJ repair, and two events from the same parent producing a 171bp insertion at AGAP007280, most parsimoniously explained by an incomplete homing event that was resolved using homology between the gRNA sequence in the construct and its cognate target in the genome (See Supp Fig 4). Consistent with rare incomplete homing events generating a non-functional homing allele, we recovered an identical event in a single individual that produced progeny with a normal Mendelian segregation of the transgenic phenotype.

Though homing rates were high in the germline of both males and females, the fertility of females heterozygous for a homing construct was markedly reduced, with the number of larvae produced only 4.6% of wild-type (bootstrap 95% confidence limits 2.3–7.7%) for AGAP011377 and 9.3% (5.7–14.2%) of wild-type for AGAP007280. We did not recover a single larva from females heterozygous for a *CRISPR^h^* allele at AGAP005958 (Fig 2D). In contrast, *CRISPR^h^* alleles showed normal fertility in equivalent crosses of heterozygous males (Fig 2E). The fertility reduction observed for heterozygous *CRISPR^h^* females was at odds with the phenotype observed in heterozygous docking line females where the disruption of single alleles of *AGAP011377*, *AGAP007280* and *AGAP005958* apparently did not affect female fertility. This reduction in fertility is probably due to somatic expression of the Cas9 nuclease, as we have observed for a similar construct targeting GFP (Supp Fig.5), and as others have observed in *Drosophila* 9, 20. In *Drosophila*, the *nos* promoter has recently been found to be substantially more germline specific in directing Cas9 activity 20 and our system is flexible to accommodate alternative promoters.

Our measures of homing rates and fertility effects can be used with the model of Deredec et al. (2008) to derive an initial prediction about whether the constructs would be expected to spread if released into a population. This analysis revealed that the fitness cost in terms of reduced reproductive capability imposed by the *CRISPR^h^* constructs at *AGAP01377* and *AGAP005958* outweigh the homing rate, and the constructs would be expected to disappear from a population over time – in many aspects these constructs match the requirements of female-specific RIDL (Release of Insects with a Dominant Lethal) with enhanced transmission 21, a potent form of the Sterile Insect Technique, though conditional rescue of the sterility may be required for efficient production However, the higher homing rates observed for *CRISPR^h^* at *AGAP007280* combined with the milder fertility reduction observed in heterozygous females indicate that this construct could spread through a population, at least initially, and impose a reproductive load on the population as it did so, fulfilling one of the major requirements for a functional gene drive measure for vector control (Fig. 3A). To investigate the ability of the *CRISPR^h^* allele to spread at the AGAP007280 locus, caged populations were initiated with CRISPR^h^/+ and wild type individuals at equal frequency and monitored over several generations. Consistent with the modelling predictions we observed a progressive increase in the frequency of individuals positive for the *CRISPR^h^* allele from 50% to 75.1% over 4 generations (Fig. 3B). Such a reproductive load will impose a strong selection pressure for resistant alleles, some of which will be generated by the system itself through NHEJ repair of endonuclease-induced chromosome breaks, as we previously showed molecularly (Supp. Fig 4). The longer term dynamics will depend on the efficiency of spreading on the one hand and the fitness cost of mutations arising at the cleavage site on the other hand^10^, ^22^. Ultimately the effect of these mutations can be mitigated by designing nucleases that target conserved, functionally constrained regions in the desired gene and that are tolerant of mutations^1^. In the case of CRISPR this can be achieved by the use of multiple guide RNAs targeting sequence variants^7^.

The high frequency with which gene knockouts were achieved at three separate loci, and the ease with which these could be both tracked using a visual marker and secondarily modified to include genes of choice [AU do you mean using gene editing, if so please say here], establishes a reproducible tool for gene editing that will be extremely valuable for functional genetics in the malaria mosquito. The rates of super-Mendelian inheritance that we observe with CRISPR-based homing constructs at female fertility loci establish a solid basis for the development of a gene drive system that has the potential to substantially reduce mosquito populations. Moreover our gene drive element was able to carry substantial additional sequence in the form of the RFP marker unit, indicating that this technology is also resilient to bringing along additional cargo, making it suitable for population modification strategies aimed at modifying vector populations with transgenes conferring useful phenotypes such as parasite-resistance. AU briefly say what the next steps are to improve the gene drive tech. Being able to use CRISPR-Cas9 in mosquitoes means that genome editing and nuclease engineering will no longer be technical bottlenecks in this major pest insect.

The success of gene drive technology for vector control will depend on the choice of suitable promoters to effectively drive homing during the process of gametogenesis, the phenotype of the disrupted genes, the robustness of the nuclease during homing and the ability of the target population to generate compensatory mutations.

## Online Methods

### Choice of Target Genes

#### Sterility Index – p(sterile)

To assess the likely effects on sterility as a result of gene inactivation we created a sterility index with logistic regression models in *Drosophila* on the basis of gene expression and the correlated effects of genetic knock before applying model parameters to the *Anopheles* genome. The models were analysed with the R statistical programming language (www.r-project.org).

##### Gene Expression

MozAtlas 11 and FlyAtlas 23gene expression estimates were obtained for both *Anopheles* and *Drosophila* probe sets. In order to make *Anopheles* gene expression comparable with *Drosophila*, sex-specific samples were pooled together and the maximum intensity recorded in either sex. If multiple probes were present for a gene, expression in each tissue was calculated as maximum probe intensity, while probes present in multiple genes were omitted from further analysis. Only probes indicating expression as ‘present’ in at least 3of 4 biological replicates were included in this analysis. Models were constructed on the basis of rank normalized gene expression in the Head, Carcass, Testis and Ovary of *Drosophila* gene expression. For each tissue, gene expression was ranked from lowest to highest expression intensity (ties were allocated the minimum rank for that group of genes) and divided by the number of genes in the dataset. Rank normalized values fall between 0 and 1, which reflect the proportion of genes with a lower expression value in that tissue, e.g. a value of 0.8 indicates that 80% of other genes in that tissue have a lower expression level. Tissue specificity was represented by the tau-statistic 24. Expression was normalized in each tissue against maximum expression for that gene. These values are divided by the number of tissues (n-1) and subsequently summed together. The value will lie between 0 and 1. A value of 1 equals specific expression. A value of 0 is equal to ubiquitous expression.

##### Phenotype Annotations

Phenotype annotations were obtained from FlyBase (2011_7) 25 (Supp Table 1). For modelling, annotations were accepted if the associated gene had either more than 10 alleles or evidence of a null sterile annotation. Specifically, genes with more than 10 alleles, but not annotated as sterile, were included in the model as NONSTERILE (n=1509). Genes annotated with a sterile identifier, and either more than 10 alleles or evidence of a null sterile mutation were included in models as STERILE (n=536). The remaining genes were left as UNKNOWN and not included in modelling (n=8886).

##### Logistic Regression

The results of the logistic regression are shown in Supp. Table 2. The product (ovary:tau) of ranked ovary expression and tissue specificity had the highest correlation with a female sterile annotation. Once we extended the coefficients obtained for *Drosophila* to the *Anopheles* expression dataset for the same tissues, we found 271 *Anopheles* genes with a p(sterile) score ≥ 0.5 (the full list is provided in Supp. Table 4). We refer to the p(sterile) value of genes as the sterility index.

### Generation of donor constructs for gene targeting via CRISPR or TALEN mediated HDR

Gene targeting vectors were assembled by Gateway cloning (Invitrogen) and designed to contain an attP-flanked 3xP3::GFP marker construct enclosed within homology arms extending 2kb either direction of the expected CRISPR^h^ cleavage site, as well as an external 3xP3::RFP marker. Regions flanking the target sites for each gene were amplified primers that included the necessary recombinase sites for the Gateway reaction (underlined). For AGAP005958: 5958-T1[5'F1]B1 (GGGGACAAGTTTGTACAAAAAAGCAGGCTGTGCAAGCTAGCCGTTTCGAG) and 5958-T1[5'R1]B4 (GGGGACAACTTTGTATAGAAAAGTTGGGTGCGCGGCTCCAGTATCTCGTCA) as well as 5958-T1[3'F1]B3 (GGGGACAACTTTGTATAATAAAGTTGAGCTGGATTTCACAATCTCCGA) and 5958-T1[3'R1]B2 (GGGGACCACTTTGTACAAGAAAGCTGGGTACTCGTGCATTTGACTGCTTCC) to generate the left and right arms of homology respectively. Regions flanking the AGAP007280 target site were amplified using 7280-T1[5'F1]B1 (GGGGACAAGTTTGTACAAAAAAGCAGGCTCAGATACTGATGCCGCAGGTTCA) and 7280-T1[5'R1]B4 (GGGGACAACTTTGTATAGAAAAGTTGGGTGGAAAGTGAGGAGGAGGGTGGTAGTG) as well as 7280-T1[3'F1]B3 (GGGGACAACTTTGTATAATAAAGTTGTTTCTTCCTCACCTCGCTGCGA) and 7280-T1[3'R1]B2 (GGGGACCACTTTGTACAAGAAAGCTGGGTACCCCTCCAGCTATGATCAACATGC) to generate the left and right arms of homology respectively. Regions flanking the AGAP011377 target site were amplified using 11377-T1[5'F1]B1 (GGGGACAAGTTTGTACAAAAAAGCAGGCTCTAGTGGCTACAGGCAGGCC) and 11377-T1[5'R1]B4 (GGGGACAACTTTGTATAGAAAAGTTGGGTGGAAATTTTCCGGCGCCAGGC) as well as 11377-T1[3'F1]B3 (GGGGACAACTTTGTATAATAAAGTTGTTTCTACGTCTGCTACAACG) and 11377-T1[3'R1]B2 (GGGGACCACTTTGTACAAGAAAGCTGGGTAGACGAGTCAACTCCAGGGCT) to generate the left and right arms of homology respectively.

The amplified left and right homology arms were cloned by BP reaction (Invitrogen) into pDONR221-P1P4 and pDONR221-P3P2 respectively. The resultant pENTR vectors were assembled into donor vectors (pHDRfp-11377; pHDRgfp-5958; pHDRgfp-7280) by LR reaction (Invitrogen) with an attP-GFP-attP pENTR vector and a destination vector containing a 3xP3::RFP marker external to the arms of the homology that should not be inserted into the target locus during a legitimate homology-directed repair event.

### Generation of CRISPR and TALEN constructs

A human codon-optimised version of the *Streptococcus pyogenes* Cas9 gene (hCas9) was amplified from pX330 (AddGene/Zhang lab) using primers containing *SalI* and *PacI* sites, SalI-hCas9-F (aacgtcgacGATCCCGGTGCCACCATGGA) and PacI-hCas9-R (aacttaattaaTTTCGTGGCCGCCGGCCTTTT). hCas9 was then sub-cloned with *SalI* and *PacI* into a vasa promoter-containing vector before cloning into a RMCE vector synthesised by DNA2.0 to contain the vasa 3’ UTR regulatory sequence and a U6::gRNA cassette containing a spacer cloning site based on Hwang et al. 26, all flanked by attB recombination sites. The U6snRNA polymerase III promoter and terminator sequences were used as described previously^17^. The resultant vector, p165, was digested with BsaI and modified to contain individual gRNA spacers by golden gate cloning of appropriately designed and annealed oligos bearing complete homology to the intended target sequence with unidirectional overhangs compatible with *Bsa*I-digested p165. The full sequence of vector p165 has been deposited to GenBank (BankIt ID 1873065). The resultant vectors containing gRNAs targeting AGAP011377 (GCAGACGTAGAAATTTTC), AGAP005958 (GAGATACTGGAGCCGCGAGC) and AGAP007280 (GGAAGAAAGTGAGGAGGA) were named p16503, p16505 and p16501 respectively. Individual gRNA target sites were identified and assessed for off-targets using both the ZiFiT (http://zifit.partners.org/) and ChopChop (chopchop.rc.fas.harvard.edu) websites.

TALEN binding sites targeting the AGAP011377 gene were selected using the TALE-NT software 8 and the site TCGAAAACACGGGCctggcgccggaaaatTTCTACGTCTGCTAC was chosen to cleave at AGAP011377 at a site that overlapped with the corresponding CRISPR site (underlined) in the same gene (See Supp. Figure 7). TALEN expressing plasmids were assembled by Golden Gate cloning as described 8 using the GoldyTALEN scaffold as destination vector 27. Subsequently, each TALEN monomer was cloned into an Anopheles expression vector under the expression of the Vas2 promoter and 3’UTR and the FoKI cleavage domains were modified to be active as obligate heterodimer (DD/RR variants).

The location of the TALEN and CRISPR recognition sites in relation to the coding sequence of the target genes is shown in Supp. Figure 6. Each recognition sequence is followed by the obligatory PAM sequence of 5’-NGG distal to the region of complementarity in the gRNA sequence.

### Microinjection of mosquito embryos and selection of transformants

Freshly laid embryos of the *Anopheles gambiae* G3 strain, herein referred to as wild type, reared under standard conditions of 70% relative humidity at 26± 2 °C were used for microinjections as described elsewhere^28^.

For the generation of the HDRgfp docking lines the donor construct (300ng/ul) containing regions of homology to the relevant target locus was injected together with the relevant CRISPR plasmid (300ng/ul) for AGAP007280 (p16501) and AGAP005958 (p16505) or, for AGAP011377, plasmids expressing the left and right monomers of the TALEN (each at 300ng/ul). Surviving G_0_ individuals were crossed to wild type and positive transformants were identified under fluorescence microscopy as GFP+ larvae among the G_1_ progeny.

For the recombinase-mediated cassette exchange reactions a mix containing the relevant CRISPR plasmid (200ng/ul) and 400ng/ul vasa::integrase helper plasmid 29 was injected into embryos of the HDRgfp docking lines. Progeny from the outcross of surviving G_0_ individuals to wild type were screened for the presence of RFP and the absence of GFP that should be indicative of a successful cassette exchange event.

### Containment of gene drive mosquitoes

All mosquitoes were housed at Imperial College London in an insectary that is compliant with Arthropod Containment Guidelines Level 2 30. All GM work was performed under institutionally approved biosafety and GM protocols. In particular GM mosquitoes containing constructs with the potential to show gene drive were housed in dedicated cubicles, separated by at least 6 doors from the external environment and requiring two levels of security card access. Moreover, because of its location in a city with a Northern temperate climate, *Anopheles gambiae* mosquitoes housed in the insectary are also ecologically contained. The physical and ecological containment of the insectary are compliant with guidelines set out in a recent commentary calling for safeguards in the study of synthetic gene drive technologies 18.

### Molecular confirmation of gene targeting and recombinase-mediated cassette exchange

**Figure 1:**
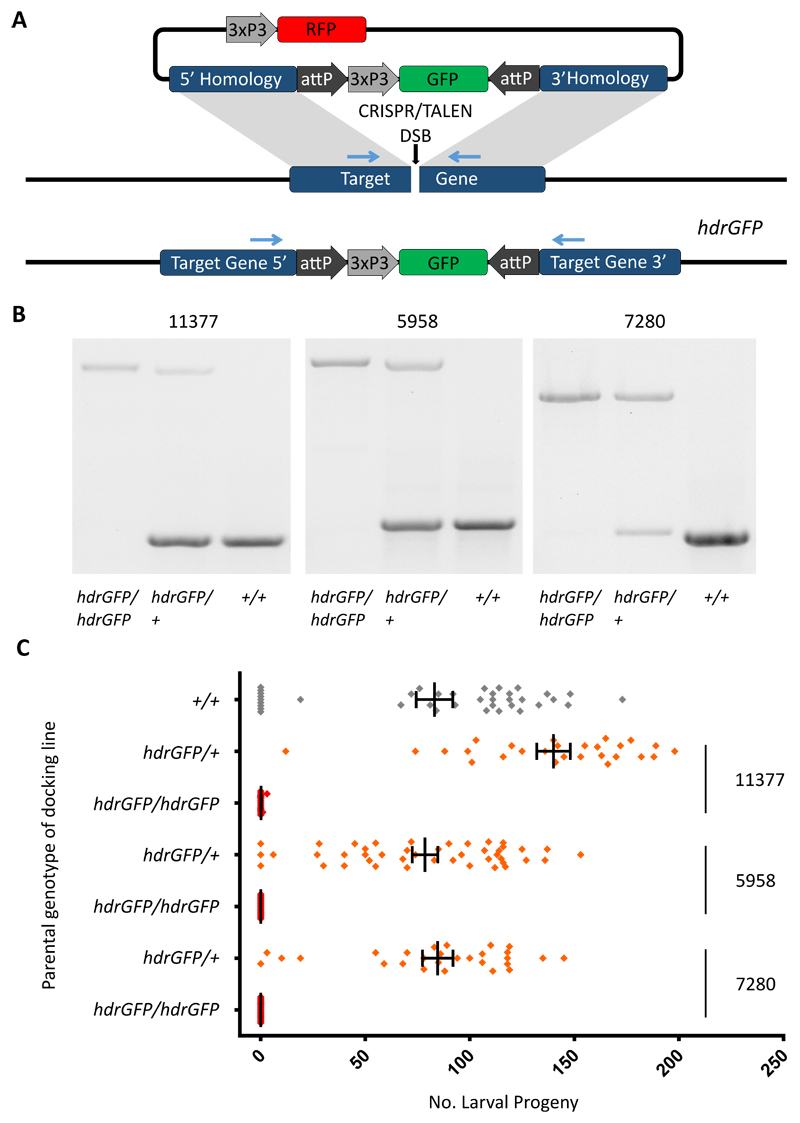
Gene disruption by homology directed repair at three separate loci causes recessive female sterility. A) A plasmid-based source of either a TALEN or Cas9 coupled with a gRNA induces a double stranded break at the target locus. A plasmid (hdrGFP) containing regions of homology immediately upstream and downstream of the cut site acts as a template for homology-directed repair. Internal to the homology regions a 3xP3::GFP cassette identifies hdrGFP integration events and two attP sites facilitate secondary modification of the locus through RMCE. B) PCR was used to confirm the targeted loci in wild type individuals as well as those homozygous and heterozygous for the *hdrGFP* allele. The primer pair used is indicated in A (blue arrows). C) Counts of larval progeny from individual females homozygous or heterozygous for *hdrGFP* alleles mated to wild type males. Heterozygous docking lines for all 3 loci showed at least full fertility compared to wild type females. A minimum of 20 individuals were tested for each line. Vertical bars represent the mean and error bars the standard error of the mean.

To confirm molecularly the successful integration of docking or CRISPR^h^ constructs into their genomic target site, genomic DNA was extracted using the Wizard Genomic DNA purification kit (Promega) from GFP+ or RFP+ G_1_ mosquitoes respectively. Docking sites were interrogated by PCR using primers binding the docking construct, 5’GFP-R (TGAACAGCTCCTCGCCCTTG) and 3’GFP-F (GCCCTGAGCAAAGACCCCAA) with primers binding the genome outside of the homology arms (as portrayed in Supp. Figure 1): AGAP11377 using DL-11377-F (GGGTGTTAACGTTCCGCCTA) and DL-11377-R (ACCCAAGACCACCCAAAGAC), AGAP005958 using DL-5958-F1 (CGGACACGCGGAAGTCTGAA) and DL-5958-R1 (CGACTTTCCCGGAACATTTACCA), and AGAP007280 using DL-7280-F1 (AGCACGTGCCGGCTAAAGCT) and DL-7280-R1 (GCCACACCAGCAACAGCCTTATC). For the purposes of producing a shorter amplicon that could reliably amplify both the wild type allele and the hdrGFP allele in the same PCR reaction (e.g Figure 1) the following primer pairs were used: AGAP011377 (Seq-11377-F (AACCGACAGTCCATCCTTGT) and Seq-11377-R (GAGCGTCTTTCGACCTGTTC)); AGAP007280 (Seq-7280-F (GCACAAATCCGATCGTGACA) and Seq-7280-R (CAGTGGCAGTTCCGTAGAGA)); AGAP005958 (Seq-5958-F (GCACTCGTCCGCGTTCTGAA) and Seq-5958-R (TTTGTGCTGGTGTCCGCGCT)).

Successful cassette exchange of CRISPR^h^ alleles was interrogated by PCR using primers binding the CRISPR^h^ construct, RFP2q-F (GTGCTGAAGGGCGAGATCCACA) and hCas9-F7 (CGGCGAACTGCAGAAGGGAA) with primers binding the genome: AGAP011377 using Seq-5958-F and Seq-5958-R, AGAP005958 using Seq-5958-F and Seq-5958-R, and AGAP007280 using Seq-7280-F and Seq-7280-R.

### Molecular confirmation of CRISPR activity at target loci

To assess molecularly the activity of CRISPR at the target locus, the target site was sequenced in those progeny (RFP-) that apparently failed to receive a CRISPR homing allele from a hemizygous RFP+ parent. Genomic DNA was extracted using the Wizard Genomic DNA purification kit (Promega). Amplicons 2.5kb either side of the CRISPR^h^ target site in AGAP011377, AGAP005958 and AGAP007280 were amplified with Phusion HF polymerase (Thermo Scientific) using Seq-11377-F and Seq-11377-R, Seq-5958-F and Seq-5958-R, and Seq-7280-F and Seq-7280-R primers (described above), respectively. PCR products were purified (Qiagen PCR purification kit) and sequenced using internal primers, Seq-11377-F2 (TCGCCATGTACGCCACCAAC), Seq-5958-F2 (CTTGCCGCTGCGCAGATGTT) and Seq-7280-F2 (TCCGGTGGACCGTTTGTGTG).

### Fertility assays

Heterozygous “docking line” individuals were inter-crossed to generate heterozygous and homozygous knock-in mutants. Offspring were screened for homozygous or heterozygous knock-in mutations by “strong” or “intermediate” intensity of GFP fluorescence respectively. Male and female individuals from each screened homozygous and heterozygous class were mated to an equal number of wild-type mosquitoes for 5 days. Females were blood fed on an anaesthetized mouse on the 6^th^ day and a minimum of 40 mosquitoes were isolated individually into 300ml beakers and allowed to lay 3 days later into a 25ml cup filled with water and lined with filter paper^31^. For each female, eggs and larvae were counted and 16 larvae were screened for GFP expression using a Nikon inverted fluorescence microscope (Eclipse TE200) to confirm parental hetero/homozygosity at the HDR locus. Heterozygotes were confirmed by the presence of GFP- progeny. Females that did not give larvae were dissected and checked under microscopy for the presence of sperm in their spermathecae. Those which were unmated were excluded from the phenotypic analysis.

To confirm HDR hetero/homozygosity in transgenic females which were mated but failed to give progeny, a PCR was performed across the HDR locus using a primer pair designed to amplify both the wild type allele (1kb) and the HDR+ allele (2.5kb). The following primer pairs were used for the 3 genes: AGAP011377 using Seq-11377-F (AACCGACAGTCCATCCTTGT) and Seq-11377-R (GAGCGTCTTTCGACCTGTTC), AGAP005958 using Seq-5958-F (GCACTCGTCCGCGTTCTGAA) and Seq-5958-R (TTTGTGCTGGTGTCCGCGCT), and AGAP007280 using Seq-7280-F (GCACAAATCCGATCGTGACA) and Seq-7280-R (CAGTGGCAGTTCCGTAGAGA).

Fertility assays were performed using CRISPR^h^ hemizygotes essentially the same as the “docking line” phenotype assays with the exception that 50 progeny from each parent were screened for the presence of an RFP-linked CRISPR^h^ allele to assess the frequency of CRISPR^h^ transmission.

### Ethics statement

All animal work was conducted according to UK Home Office Regulations and approved under Home Office License PPL 70/6453.

### Cage experiments

L1 mosquito larvae heterozygous for the *CRISPRh* allele at AGAP007280 were mixed within 12 hours of eclosion with an equal number of age-matched wild-type larvae in rearing trays at a density of 200 per tray (in approx. 1L rearing water). The mixed population was used to seed two starting cages with 600 adult mosquitoes each. For four generations, each cage was fed after 5 days of mating, and an egg bowl placed in the cage 48h post bloodmeal to allow overnight oviposition. After allowing full eclosion a random sample of offspring werescored under fluorescence microscopy for the presence or absence of the RFP-linked *CRISPR^h^* allele, then reared together in the same trays and 600 were used to populate the next generation.

## Supplementary Material

Supplementary Figure 1-13

Supplementary Table 1

## Figures and Tables

**Figure 2:**
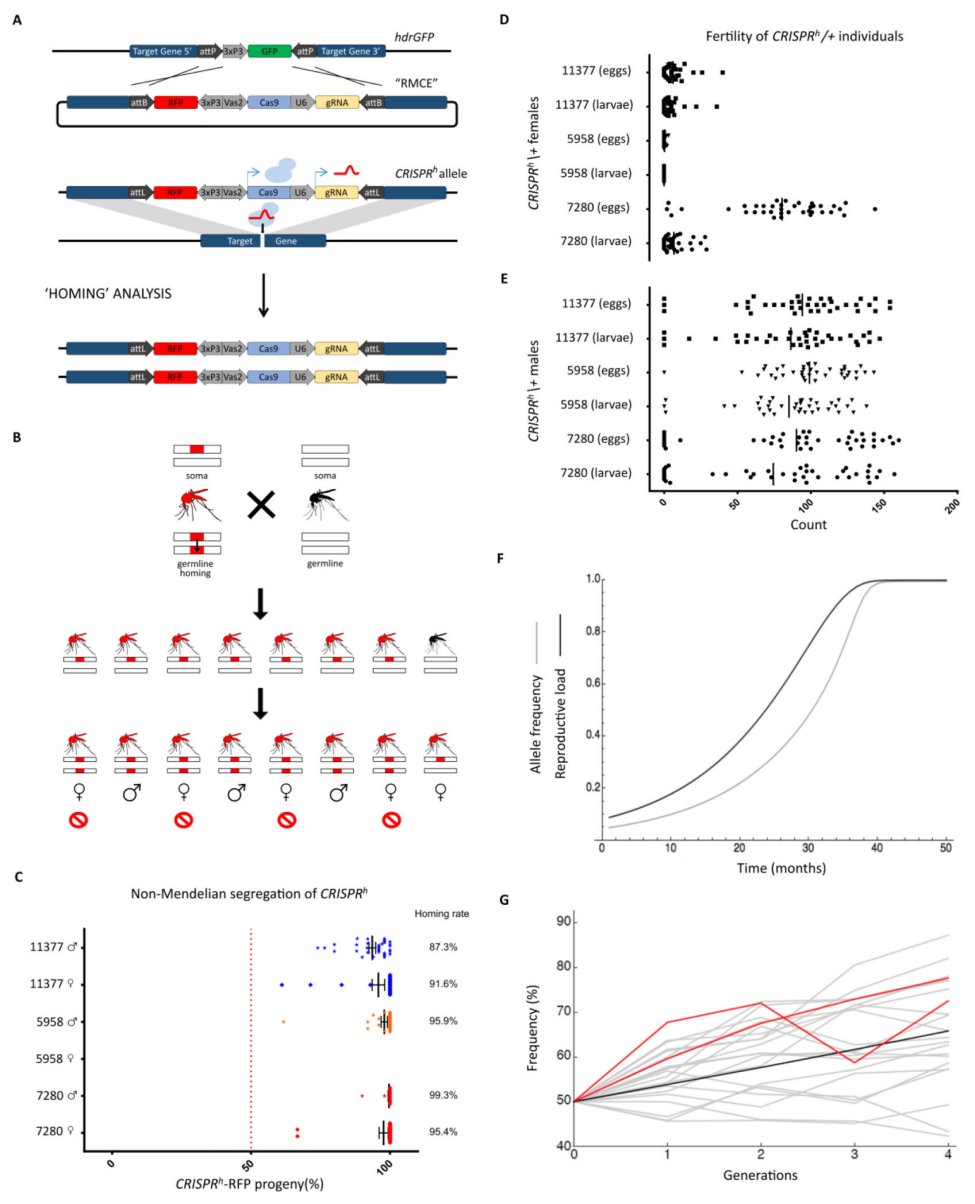
*CRISPR^h^* alleles inserted at female fertility loci show highly efficient gene drive and can spread in a caged population Recombinase-mediated cassette exchange (RMCE) was used to replace the GFP transcription unit in hdrGFP docking lines with a CRISPR homing construct (CRISPR^h^ consisting of a *3xP3::RFP* marker, Cas9 under the transcriptional control of the *vasa2* promoter and a gRNA under the control of the ubiquitous *U6* PolIII. The gRNA cleaves at the non-disrupted wild type allele. Repair of the cleaved chromosome through HDR leads to copying of the *CRISPR^h^* allele and homing. B) Confinement of homing to the germline should lead to super-Mendelian inheritance of a homing construct (indicated in red) that, when targeting a haplosufficient somatic female fertility gene, will reduce the number of fertile females. C) High levels of homing at all three female fertility loci were observed. Male or female *CRISPR^h^/+* heterozygotes were mated to wild type. Progeny from individual heterozygous females were scored for the presence of the RFP linked to the CRISPRh construct. A minimum of 35 females were analysed for each cross. The average rate of RFP+ individuals recovered per mated female is shown as a percentage. D) and E) counts of eggs and hatching larvae for the individual crosses revealed a strong fertility effect in heterozygous *CRISPR^h^/+* females (D) that was not revealed in equivalent heterozygous males. F) Dynamics calculated using recurrence equations (15) in Deredec et al. (2008), using the observed homing rates in males and females and effects on female fertility. We assume no fitness effects in males and that the initial release consists of heterozygous males equal to 10% of the pre-release adult male population (i.e. 5% of the overall population). The model assumes discrete generations (one per month) and random mating, and does not account for evolution of either the CRISPR allele or the target sequence. G) Increase in frequency of *CRISPR^h^* allele in cage population experiments. An equal number of *CRISPRh/+* and wild type individuals were used to start a population and the frequency of individuals containing a *CRISPR^h^* allele was recorded in each subsequent generation. Black line shows deterministic prediction based on observed parameter values (homing rates 98.4%, heterozygous female fitness of 9.3%, homozygous females completely sterile), assuming no fitness effects in males. Grey lines show results from 20 stochastic simulations assuming 300 males and 300 females are used to start the next generation, females mate randomly with a single male, and 15% of females fail to mate, using random numbers drawn from the appropriate multinomial distributions. Red line shows results from 2 replicate cages.

**Table 1:** Recombinase Mediated Cassette Exchange to insert CRISPR^h^ alleles at their target locus

Target Gene	Injected Eggs	G_0_ Crossed	% Founders	% cassette exchange progeny	Crosses of fertile G_1_ containing CRISPR^h^ allele			
					G1 cross	G_2_ progeny		transmission rate
						CRISPR^h^ +	CRISPR^h^ -	
AGAP007280	540	56	≥7.1% (4/56)*	0.38% (15/4000)	1 ♀G1 x wt	34	2	94.4%
					8 ♂G1 x wt	666	3	99.6%
AGAP011377	500	21	≥4.8% (1/21)*	0.13% (4/2990)	1 ♀G1 x wt	35	0	100.0%
AGAP005958	400	49	≥2.0% (1/49)*	0.05% (2/4000)	1 ♂G1 x wt	236	0	100.0%

CRISPR homing constructs were injected into the respective docking lines with a plasmid source of vasa-driven integrase. Successful recombinase-mediated exchange events were scored visually for the replacement of GFP at the docking site with the RFP contained within the CRISPR^h^ construct. The proportion of G_1_ progeny containing putative cassette exchange events is also shown.

* In these cases the progeny were screened from group crosses hence the estimate for the number of founders is a minimum.

**Table 2:** *CRISPR^h^* homing rates remain high across several generations

Line	% of progeny with *CRISPR^h^* allele in crosses to wt				Average Transmission Rate per generation	Average Homing Rate Per Generation
	G2 cross	G3 cross	G4 cross	G5 cross		
**AGAP011377 ♂+/-**	91.4% (581/636)	88.4% (1442/1631)	93.7% (1550/1654)	97.3% (491/505)	92.37%	85%
**AGAP011377**♀**+/-**	91.7% (55/60)	76.1% (70/92)	85.2% (121/142)		84.56%	69%
**AGAP005958**♂**+/-**	97.9% (1654/1689)	96.4% (268/278)			97.17%	94%
**AGAP005958**♀**+/-**	-	-			-	-
**AGAP007280**♂**+/-**	99.6% (1377/1383)	98.8% (499/505)			99.19%	98%
**AGAP007280**♀**+/-**	99.2% (255/257)				99.22%	98%

Each generation heterozygous individuals of each sex from each homing line were crossed to wild type mosquitoes and the frequency of the *CRISPR*^*h*^ allele among the progeny estimated by scoring visually for the presence of the RFP gene contained within the CRISPR^h^ construct. In all cases the progeny of the *CRISPR*^*h*^ male cross were used to maintain the line each generation. Homing rate is calculated as the percentage of wild type chromosomes converted to homed chromosomes (i.e (transmission rate-0.5)*2)

**Supplementary Table 2:** Sterility Annotations and Controlled Vocabulary (FlyBase 2011_7)

Controlled vocabulary	Genes (n)
female sterile	473
grandchildless	11
lethal | embryonic stage | female	37
lethal | embryonic stage | maternal effect	161
lethal | embryonic stage | non-rescuable maternal effect	81
lethal | embryonic stage | rescuable maternal effect	66
lethal | maternal effect | recessive	41
amorphs (null annotations)	280

**Supplementary Table 3:** Logistic Regression Coefficients

	Estimate	Z value	P value
Intercept	-1.318	-8.315	< 2e-16 *
Head	1.705	5.152	2.58e-07 *
Testis	0.535	2.066	0.039 *
Ovary	-0.833	-1.526	0.127
Tau	0.108	0.207	0.835
Ovary:Tau	-2.219	-2.865	0.004 *

**Supplementary Table 4:** P(sterile) of Target Genes

Gene	Ortholog in Drosophila	P(sterile)
AGAP007280	*nudel*	0.70
AGAP005958	*yellow-g*	-*
AGAP011377	None identified	0.61

* high variability in the expression of this gene between replicates in the original Baker et al experiment meant that it failed to meet the minimum criterion of being present in at least 3 of 4 biological replicates in order to be included in the p(sterile) analysis. RT-PCR analysis using primers specific to AGAP005958 revealed a relatively short window of expression (~6hrs at around 40 hours postbloodmeal) that was ovary-specific (data not shown).

## Editors summary

Development of a CRISPR/Cas9-based gene drive system in *Anopheles gambiae*, the main vector for the malaria parasite, paves the way for control of this pest insect.
