# Supplementary material for: A CRISPR-Cas9 Gene Drive System Targeting Female Reproduction in the Malaria Mosquito vector Anopheles gambiae
Source: Nat Biotechnol. Author manuscript; Available in PMC 2016 Jul 1. (PMC4913862; doi:10.1038/nbt.3439)
Supplement: Supplementary Figure 1-13 [file NIHMS66155-supplement-Supplementary_Figure_1-13.pdf]

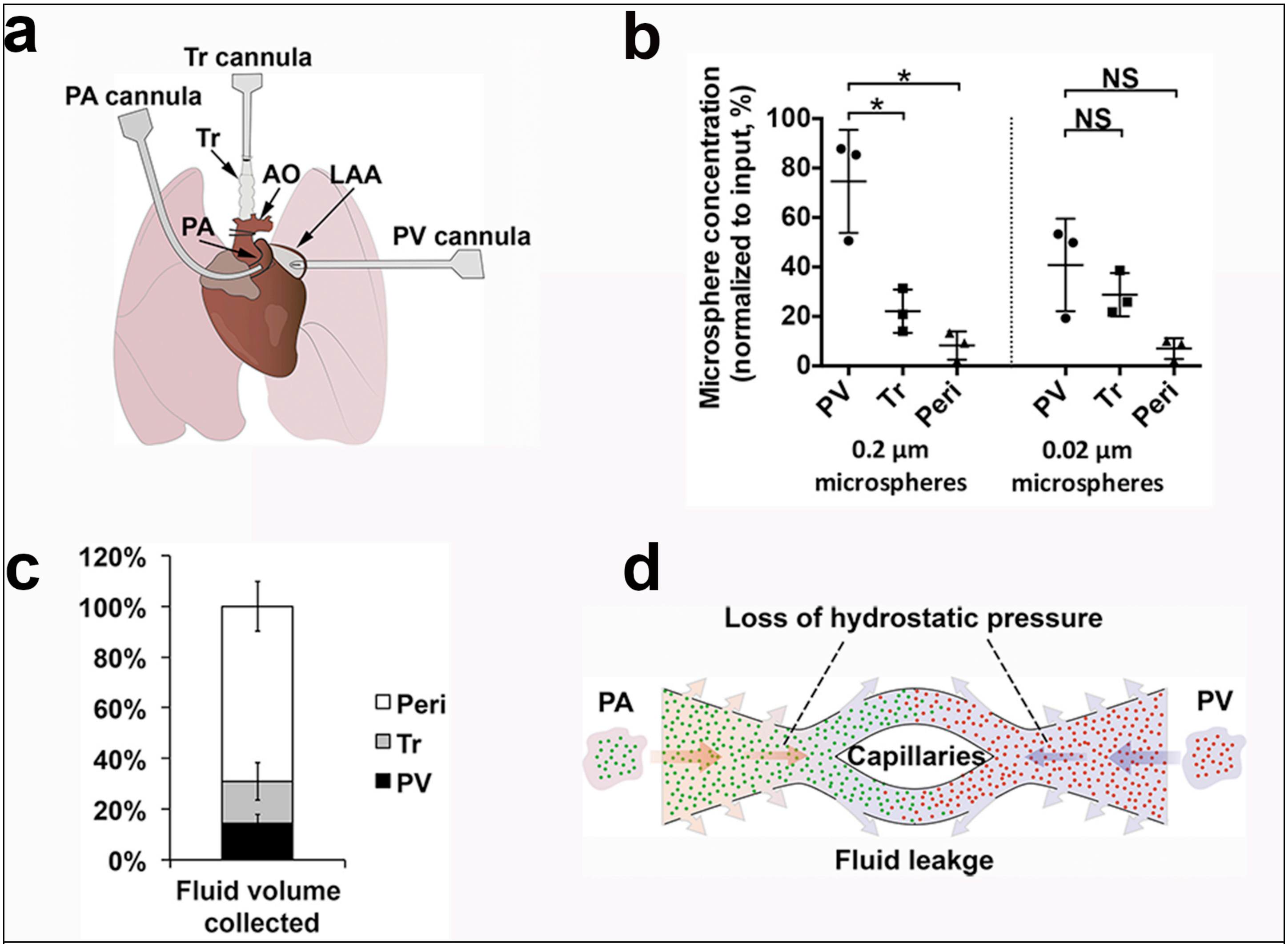

Supplementary Figure 1

Microsphere perfusion of pulmonary vascular bed in decellularized rat lungs.

(**a**) Optimization of decellularized rat lung cannulation. The PA and trachea (Tr) were directly cannulated, the PV was cannulated through the left atrial appendage (LAA), and the aorta (AO) was ligated. This cannulation strategy allowed perfusion though both the PA and PV, and fluid collection from the Tr and PV. (**b**) Concentration of 0.2-µm and 0.02-µm microspheres in the fluids collected from the PV, Tr and lung periphery (Peri) during perfusion through the PA, normalized to microsphere concentrations of the input. With PA perfusion at 40 mm Hg, 0.2-µm microspheres were collected from the PV at a concentration not significantly different from input (74.6% ± 20.8%, P = 0.17) but significantly higher than that in fluids collected from the Tr (22.1% ± 8.7%, P < 0.05) and Peri (8.2% ± 5.7%, P < 0.05). With smaller microspheres (0.02 µm), the concentration of microspheres collected from the PV (40.8% ± 18.7%) was not significantly different from that collected from the Tr (28.8% ± 8.7%, P = 0.39) or from the Peri (7.0% ± 4.2%, P = 0.08) (**c**) Fluid volumes collected from the PV, Tr and Peri during microsphere perfusion through the PA (normalized to the total volume collected from the three compartments). The majority of fluid infused into the PA drained from the Peri and Tr; only 14.5% ± 3.5% of input fluid could be collected from the PV. (**d**) A diagram showing microsphere perfusion through the PA and PV of decellularized lungs, highlighting fluid leakage along the vascular tracks and gradual reduction of hydrostatic pressure during perfusion. Error bars represented s.d. of experimental values, asterisk (*) indicated P < 0.05 and NS indicated non-significant.

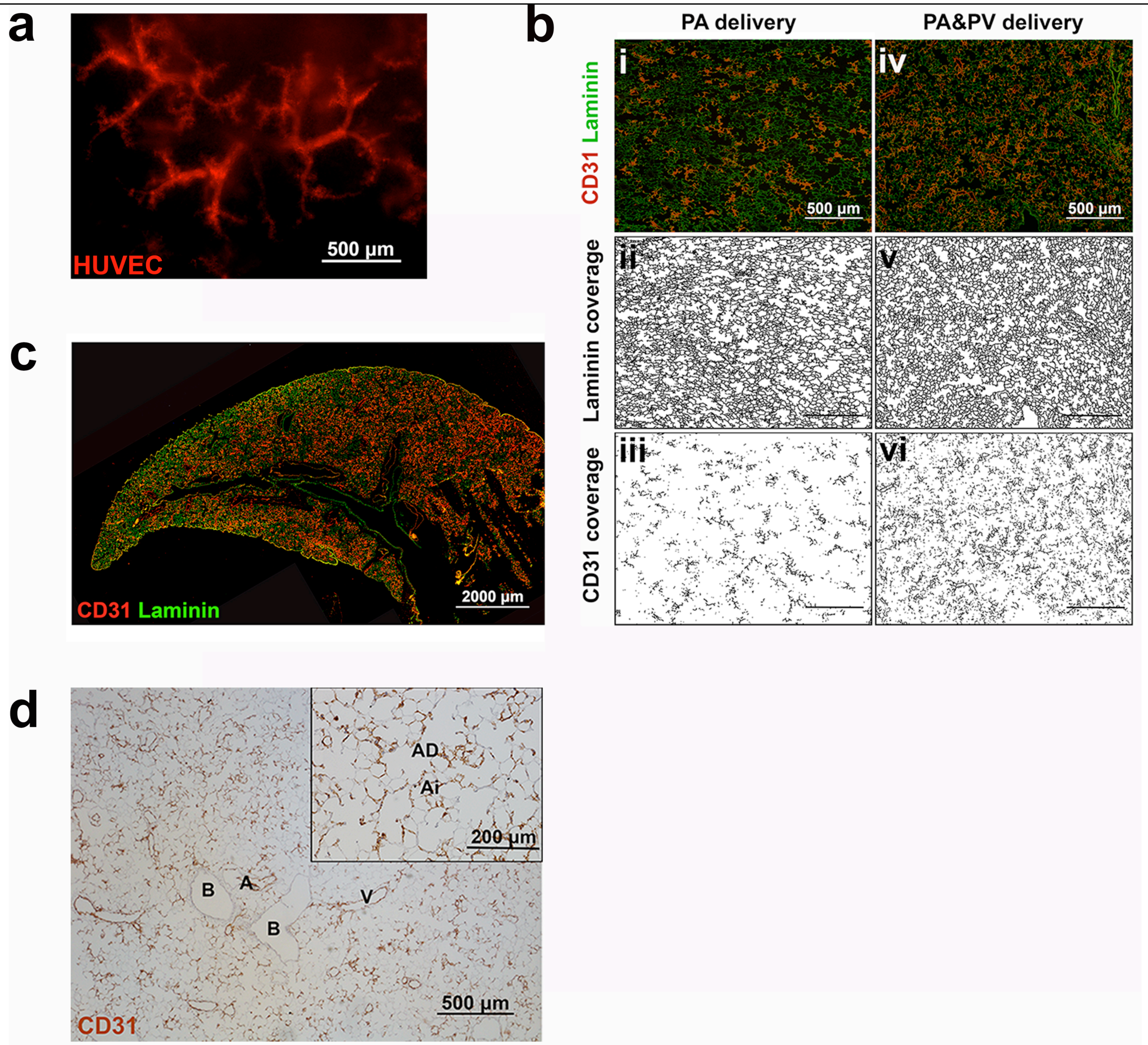

Supplementary Figure 2

Optimization of endothelial delivery into decellularized rat lungs.

(**a**) A whole-mount image of a decellularized rat lung seeded with fluorescently labeled HUVECs. (**b**) Representative images of endothelial coverage of regenerated lungs 1 d after HUVEC delivery through the PA (i,ii,iii) or through the PA&PV (vi,v,vi). Upper panel showed fluorescent images of CD31 (red, endothelial cells) and Laminin (green, lung matrix) (i,vi). Middle panel showed processed image of Laminin (from that of upper panel) for its coverage quantification (ii,v). Lower panel showed processed image of CD31 (from that of upper panel) for its coverage quantification (iii,vi). (**c**) A representative stitched image showing endothelial coverage of decellularized rat lungs 1 d after HUVEC delivery through the PA&PV (CD31, red; Laminin, green). (**d**) A representative image of CD31 immunohistochemical staining of a decellularized rat lung 1 d after HUVEC delivery through the PA&PV (CD31, brown) demonstrating well endothelialization of arteries (A) and veins (V), and absence of endothelial cells in bronchus (B). Ai, alveoli; AD, alveolar duct.

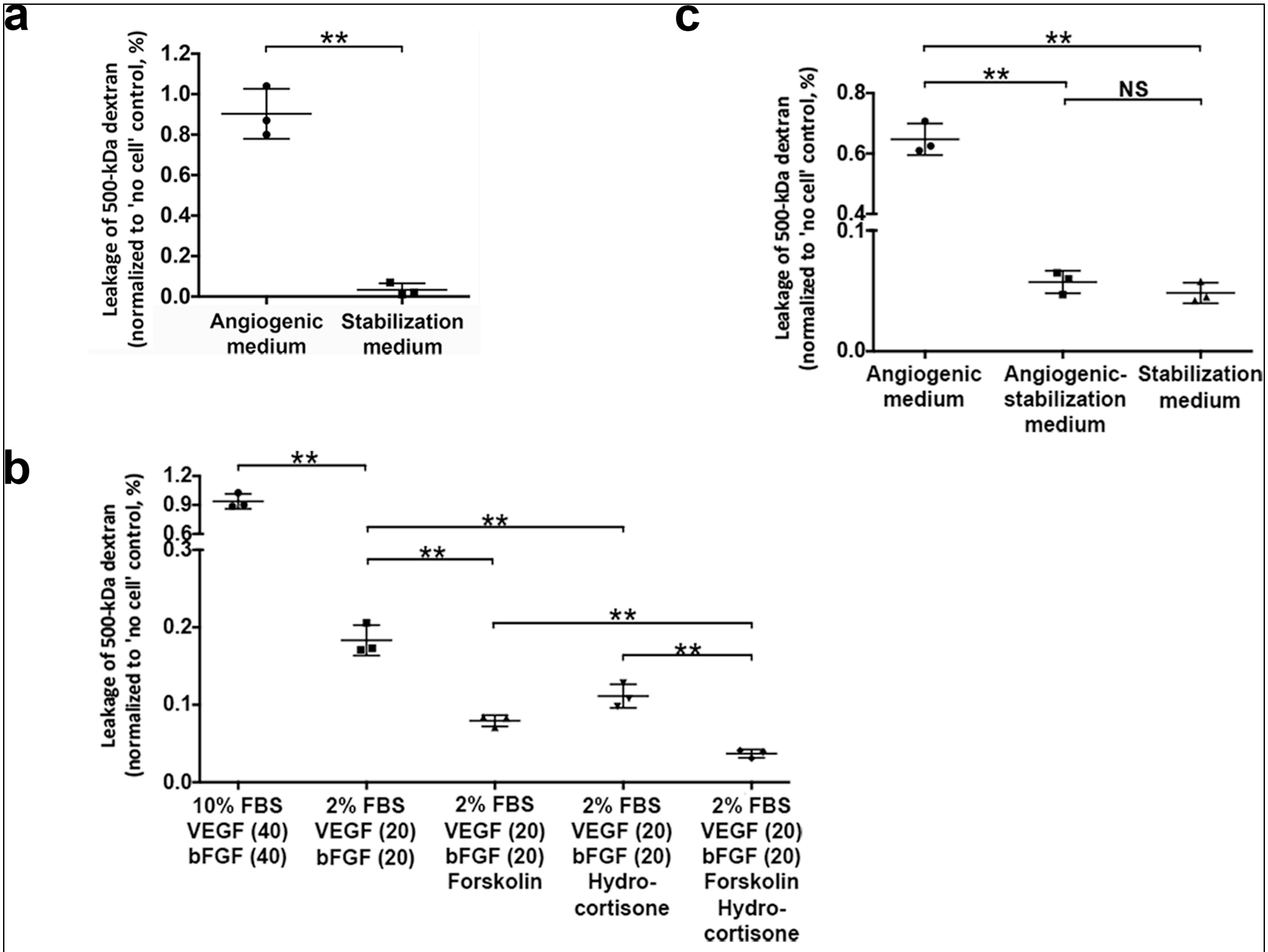

Supplementary Figure 3

Relative leakage of 500-kDa dextran in HUVEC transwell permeability assays.

(a) Relative dextran leakage through HUVEC monolayers after culture in angiogenic or stabilization medium for 2 d. (**b**) Examination of the effect of individual components in stabilization medium on dextran leakage through HUVEC monolayers after 2 d of culture. Media under testing included basal medium supplemented with 10% FSB, VEGF (40 ng/ml) and bFGF (40 ng/ml); basal medium supplemented with 2% FBS, VEGF (20 ng/ml) and bFGF (20 ng/ml); basal medium supplemented with 2% FBS, VEGF (20 ng/ml), bFGF (20 ng/ml) and forskolin (10 μM); basal medium supplemented with 2% FBS, VEGF (20 ng/ml), bFGF (20 ng/ml) and hydrocortisone (110 nM); and basal medium supplemented with 2% FBS, VEGF (20 ng/ml), bFGF (20 ng/ml), forskolin (10 μM) and hydrocortisone (110 nM). (**c**) Relative dextran leakage through HUVEC monolayers after constant exposure to angiogenic medium (4 d), after sequential exposure to angiogenic medium (2 d) and then stabilization medium (2 d), and after constant exposure to stabilization medium (4 d). Leakage values were normalized to those of transwells without HUVECs ('no cell' control). Error bars represented s.d. of experimental values, and double-asterisk (**) indicated $P < 0.01$, and NS indicated non-significant.

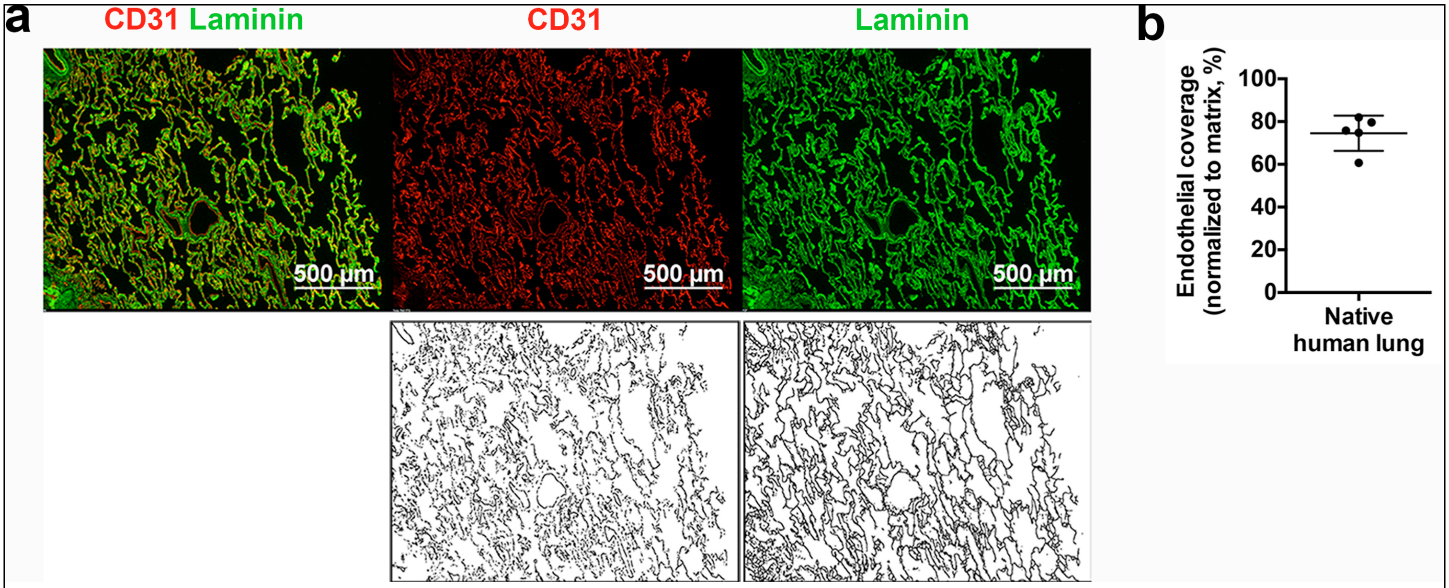

Supplementary Figure 4

Quantification of endothelial coverage in the native human lung.

(**a**) A representative image of endothelial coverage of the native human lung. Upper panel showed fluorescent images of CD31 (red, endothelial cells) and Laminin (green, lung matrix). Lower panel showed processed image of CD31 and Laminin for coverage quantification. (**b**) Quantification of CD31 (endothelial) coverage normalized to Laminin (matrix) coverage. Error bars represented s.d. of experimental values.

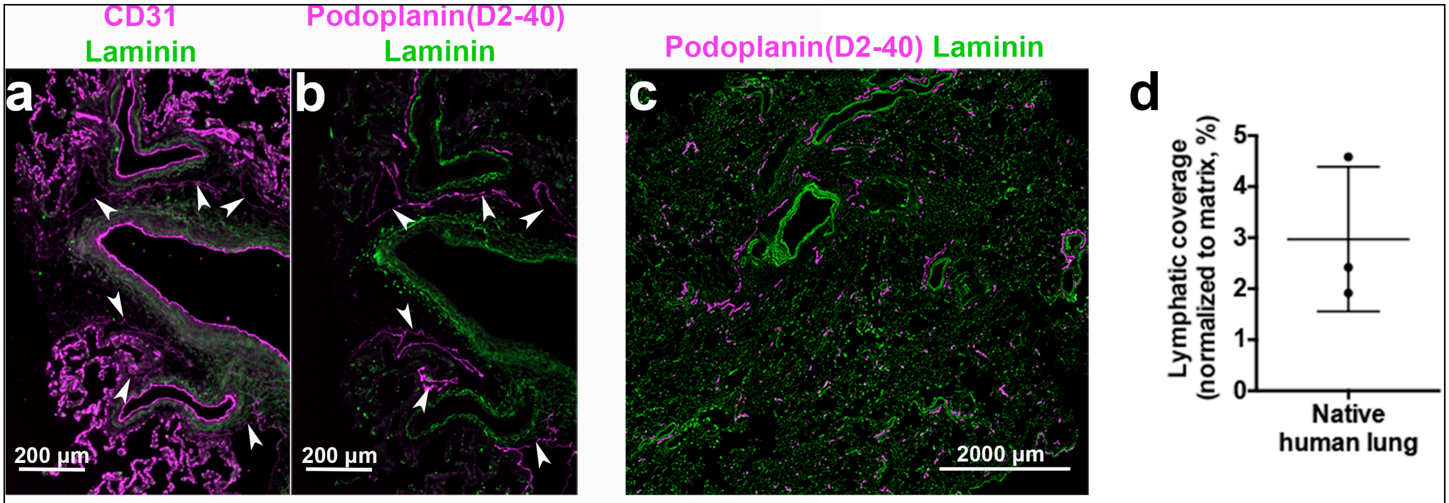

Supplementary Figure 5

Quantification of lymphatic coverage in the native human lung.

CD31 (**a**) and Podoplanin (D2-40) (**b**) staining on sequential sections of the same human lung sample (arrowheads indicated corresponding lymphatic vessels in both staining). (**c**) Large view of lymphatic distribution by a stitched image of Podoplanin (D2-40) staining. (**d**) Quantification of Podoplanin (D2-40) (lymphatic endothelial) coverage normalized to Laminin (matrix) coverage. Error bars represented s.d. of experimental values.

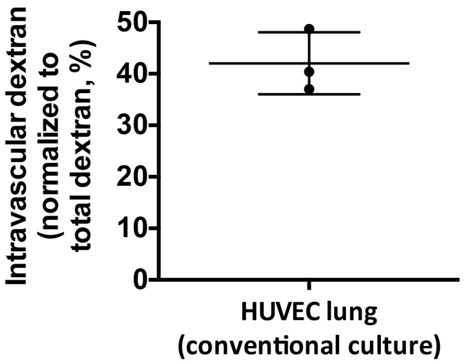

Supplementary Figure 6

Dextran perfusion and BAL assay on HUVEC lungs after conventional culture.

Quantification of intravascular dextran quantities after dextran perfusion and BAL assay on lungs seeded with only HUVECs through the PA and after 5 d of culture in conventional EGM-2 medium. Error bars represented s.d. of experimental values.

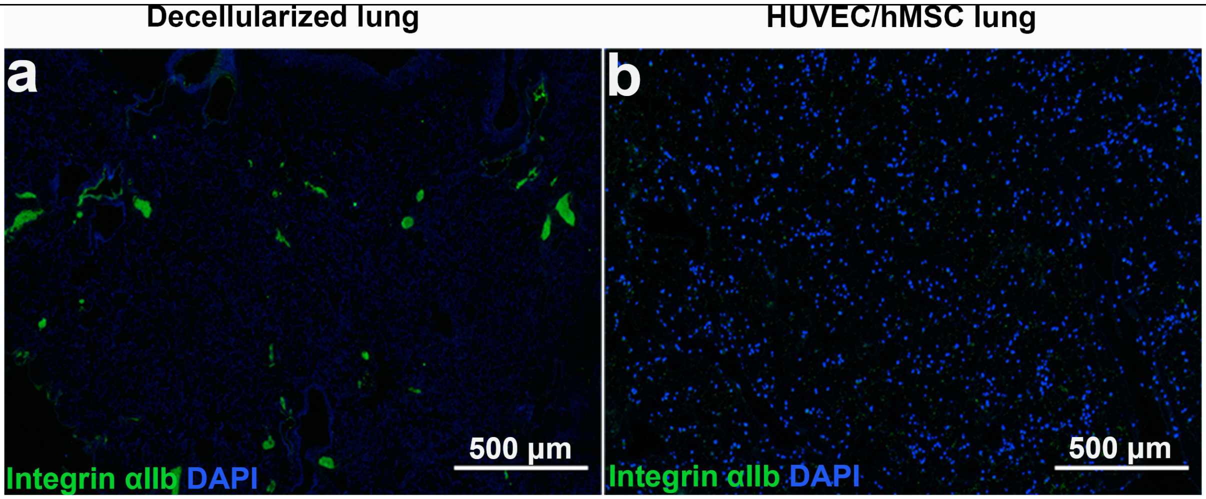

Supplementary Figure 7

Integrin αIIb and DAPI staining on decellularized and HUVEC-hMSC lungs after platelet adhesion assay.

(**a**) Integrin αIIb staining showed large number of platelet adhered and occluded the vascular bed in decellularized lungs without DAPI-positive nucleus. (**b**) Integrin αIIb staining showed only a small number of platelets in the HUVEC-hMSC lungs with reseeded cells shown by DAPI staining.

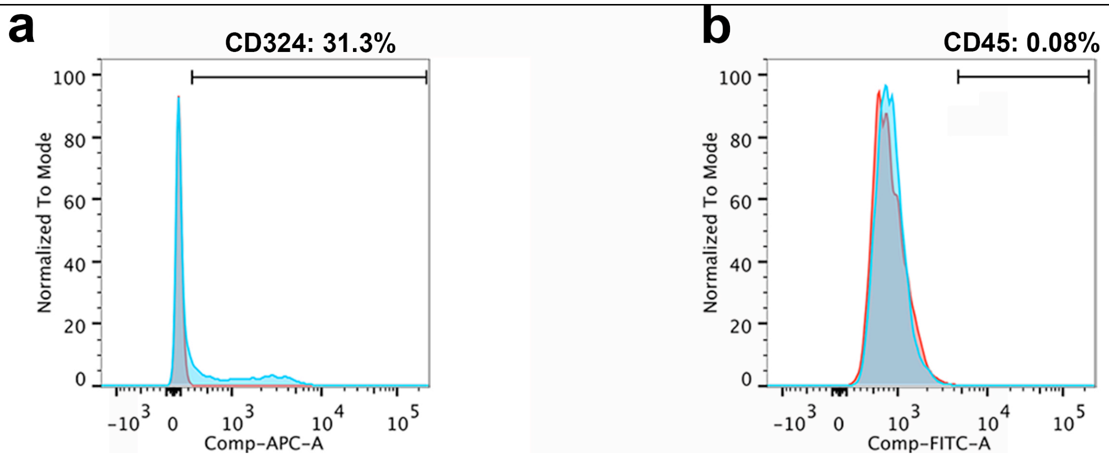

Supplementary Figure 8

Characterization of CD31^{-}CD140b^{-} cells from the endothelial differentiation of hiPSCs.

Flow cytometry analysis of CD324 (**a**) and CD45 (**b**) in CD31^{-}CD140b^{-} cells from the endothelial differentiation of hiPSCs.

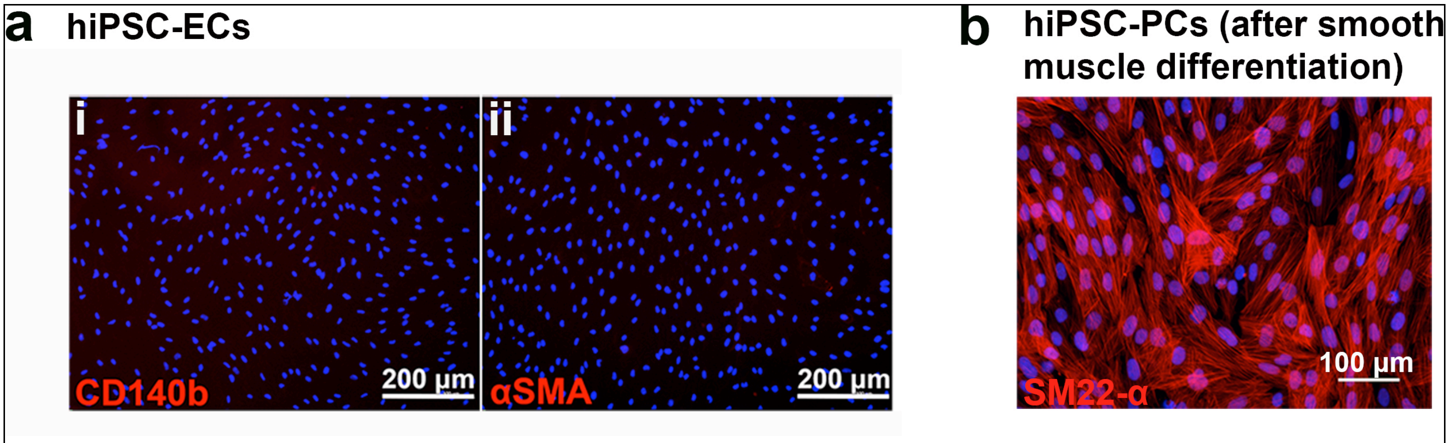

Supplementary Figure 9

Characterization of hiPSC-ECs and hiPSC-PCs.

(**a**) Absence of perivascular marker CD140b (i, red) and smooth muscle marker α-SMA (ii, red) expression in hiPSC-ECs. (**b**) Expression of SM22-α in hiPSC-PCs after smooth muscle differentiation.

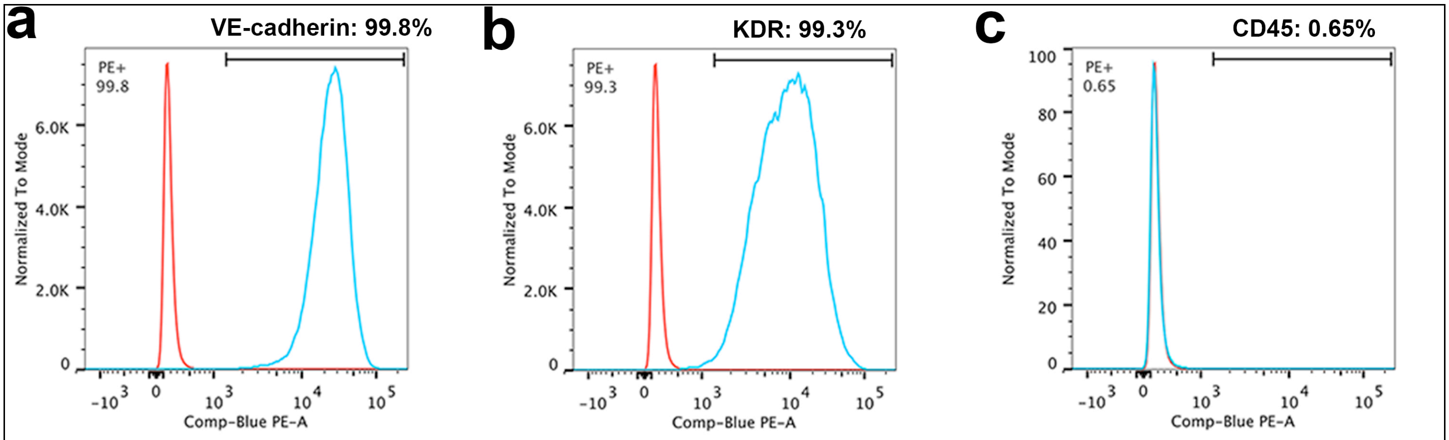

Supplementary Figure 10

Characterization of hiPSC-ECs right after FACS purification.

Flow cytometry analysis of VE-cadherin (**a**), KDR (**b**) and CD45 (**c**) in purified hiPSC-ECs, demonstrating homogenous expression of endothelial marker (VE-cadherin and KDR) and absence of hematopoietic marker CD45 expression.

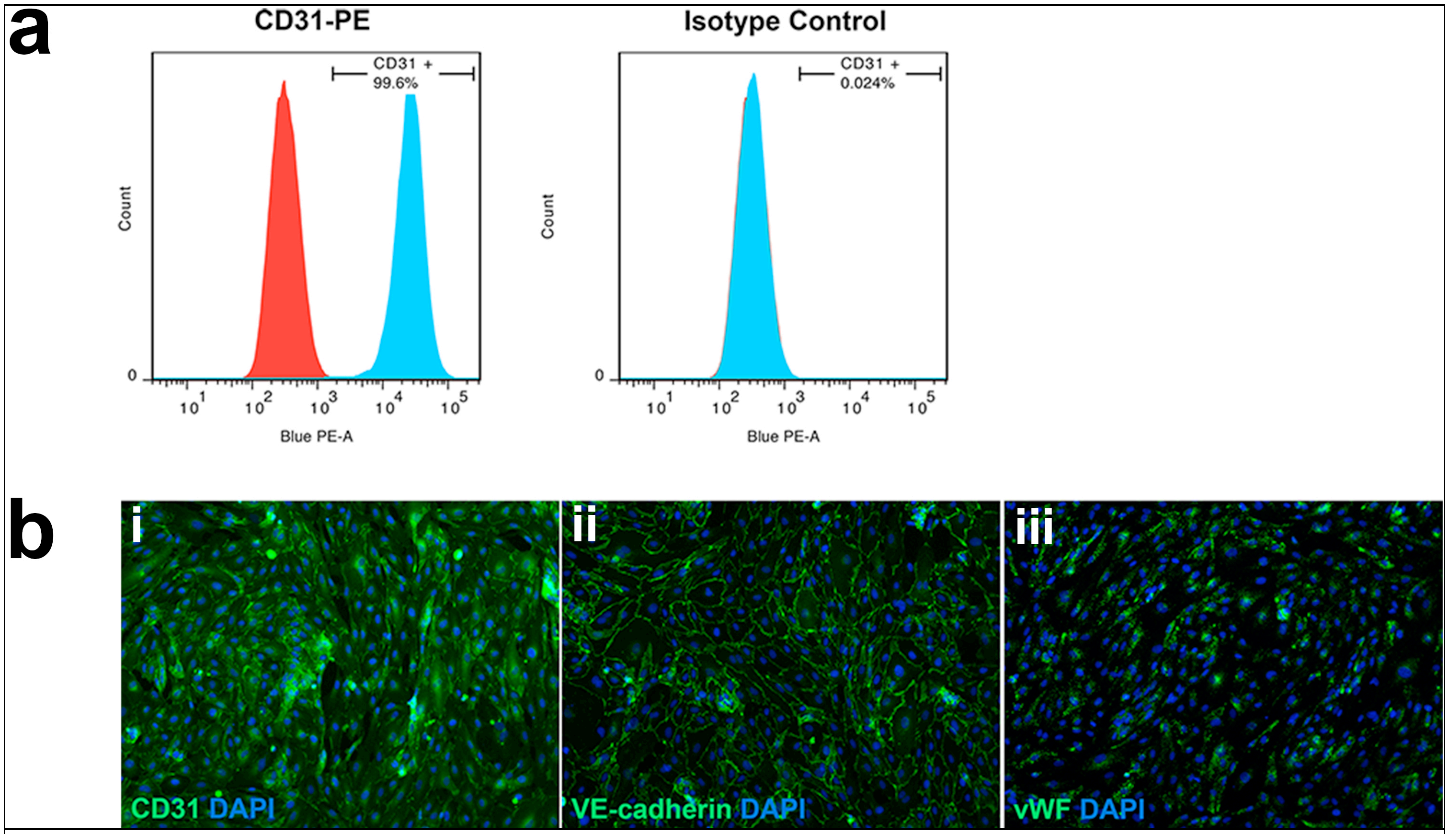

Supplementary Figure 11

Endothelial identity was maintained in hiPSC-ECs at the end of *in vitro* expansion.

Flow cytometry analysis of CD31 (**a**) and immunofluorescence staining (**b**) of CD31 (i), VE-cadherin (ii) and vWF (iii) of hiPSC-ECs at the end of expansion.

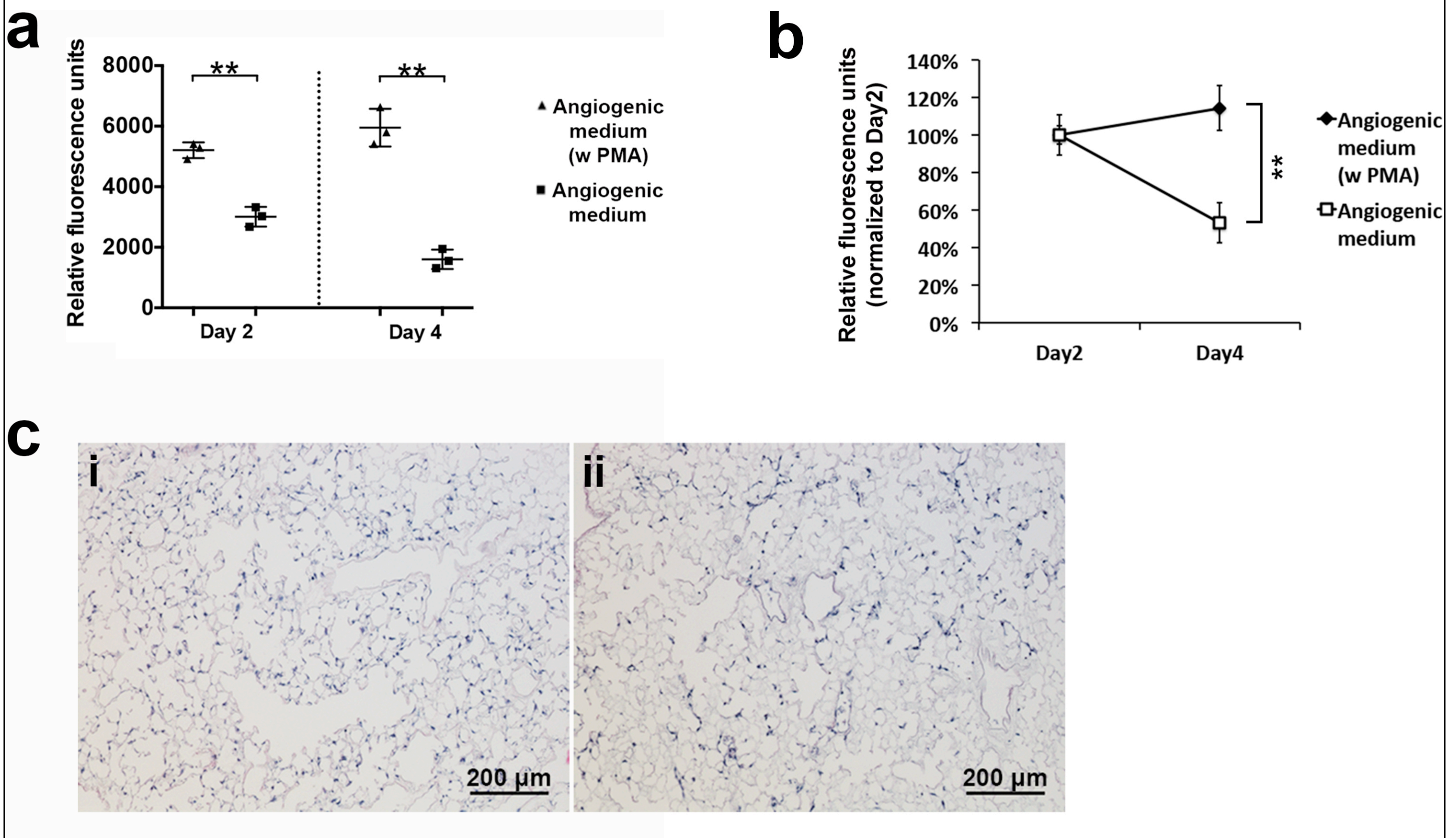

Supplementary Figure 12

Comparison of hiPSC-EC viability in decellularized lung scaffolds when cultured in angiogenic medium with and without PMA supplementation.

Relative fluorescence units of perfusate were measured after resazurin perfusion assay in lungs seeded with hiPSC-ECs on d 2 and d 4 of culture. (a) Relative fluorescence units. (b) Relative fluorescence units normalized to those on d 2. (c) Representative images of H&E staining of lungs seeded with hiPSC-ECs after 4-d culture in angiogenic medium with (i) and without (ii) PMA supplementation. Error bars represented s.d. of experimental values, and double-asterisk (**) indicated $P < 0.01$.

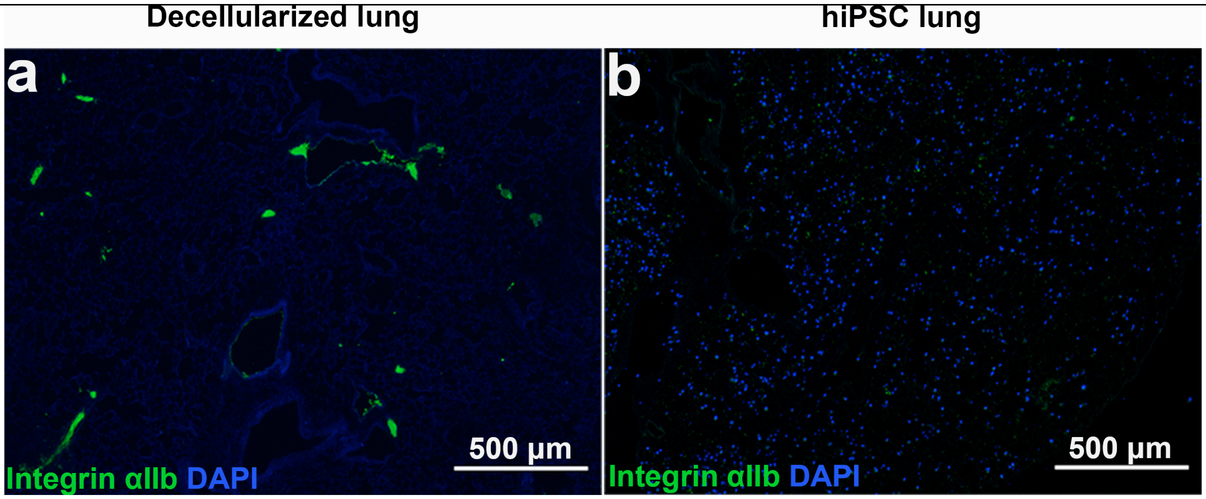

Supplementary Figure 13

Integrin αIIb and DAPI staining on decellularized and hiPSC-regenerated lungs after platelet adhesion assay.

(**a**) Integrin αIIb staining showed large number of platelet adhered and occluded the vascular bed in decellularized lungs without DAPI-positive nucleus. (**b**) Integrin αIIb staining showed only a small number of platelets in the hiPSC-regenerated lungs with reseeded cells shown by DAPI staining.
