# Supplementary material for: A CRISPR-Cas9 Gene Drive System Targeting Female Reproduction in the Malaria Mosquito vector Anopheles gambiae
Source: Nat Biotechnol. Author manuscript; Available in PMC 2016 Jul 1. (PMC4913862; doi:10.1038/nbt.3439)
Supplement: Supplementary Table 1 [file NIHMS66155-supplement-Supplementary_Table_1.docx]

**Supplementary Table 1 CRISPR or TALEN-mediated HDR to disrupt candidate female fertility genes and produce docking lines**

Each target gene was injected with a vasa-driven plasmid source of either a CRISPR or TALE nuclease, as indicated, together with a hdrGFP targeting construct containing a GFP marker gene flanked on each side by 2kb of homology immediate to the nuclease target site. G_0_ individuals surviving the injections (“founders”) were crossed to wild type mosquitoes and the progeny screened visually for the presence of the GFP gene. The proportion of G_1_ progeny showing HDR is also shown. * In these cases the progeny were screened from group crosses hence the estimate for the number of founders is a minimum.

| **Target Gene** | **Nuclease** | **Injected Eggs** | **Female Founders** | | **Male Founders** | | **Total** | |
| --- | --- | --- | --- | --- | --- | --- | --- | --- |
|  |  |  | **% Founders** | **% HDR progeny** | **% Founders** | **% HDR progeny** | **% Founders** | **% HDR progeny** |
| AGAP007280 | vasa::Cas9-U6::gRNA plasmid | 350 | 67% (8/12) | 21% (262/1250) | ≥2.8% (1/36)* | 5.6% (16/286) | ≥18.8% (9/48) | 18.1% (278/1536) |
| AGAP005958 | vasa::Cas9-U6::gRNA plasmid | 760 | ≥12.5% (2/16)* | 0.66% (13/1961) | ≥10% (1/10)* | 3.1% (38/1223) | ≥11.5% (3/26) | 1.6% (51/3184) |
| AGAP011377 | vasa::TALEN-L-DD plasmid + vasa::TALEN-R-RR plasmid | 600 | ≥14% (2/14)* | 1.1% (21/1890) | ≥7.7% (1/13)* | 0.005% (1/1911) | ≥11.1% (3/27) | 0.5% (22/3801) |
